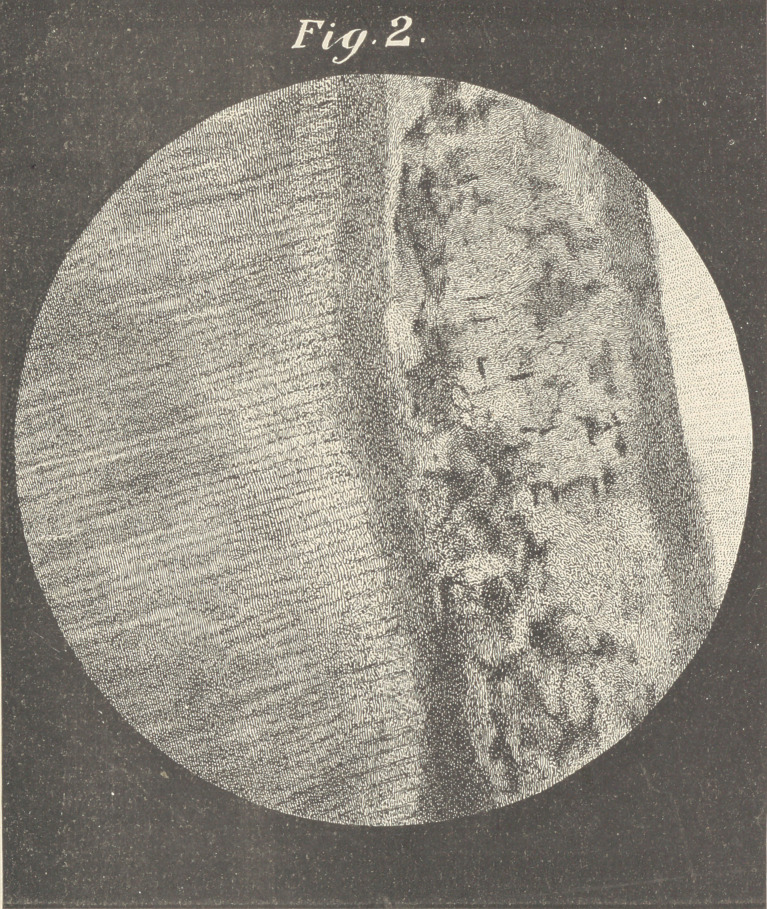# Dead and Diseased Teeth and Their Treatment

**Published:** 1888-05

**Authors:** Edward S. Niles


### DEAD AND DISEASED TEETH AND THEIR TREATMENT.

BY EDWARD S. NILES, D. M. D.

READ BEFORE THE CENTRAL DENTAL ASSOCIATION OF NORTHERN NEW JERSEY, OCTOBER 10TH, 1887.*

The subject upon which I have asked your attention this evening embraces one of the departments of our specialty which, during the past few years, has come from darkness into light, for, to the best of my knowledge, the treatment of teeth that had been de-

* Re-written and given in a lecture at the Maryland University Dental School.

prived of their central nerve and blood supply revealed little light or knowledge of the difficulty to be overcome. This was especially apparent in the prescribed treatment of abscessed teeth of long standing. Cold alveolar abscess, or "gum boils," as long as free from pain, have in the years past been considered by the dentist and patient of little importance. Two years ago, one of the most eminent of our profession in a dental meeting said, that "in his experience, he had found that it was better to let these teeth alone; if disturbed, inflammation and suppuration will follow."

Those who have attempted any course of treatment have been few, and the results must be suggested by the medicines and methods resorted to; the dressings advocated, for instance, are aromatic sulphuric acid, hydro-chloric acid, chloride of zinc, creosote, carbolic acid, salicylic acid and wine of opium. It will be seen that, with the exception of the last named, all these agents have a destructive power on soft, and some of them on hard tissues, and as from time to time they are applied, inflammation and breaking down of tissues, discharge of pus and serum follows. I once saw this state of things continue for weeks and months, while the good old practitioner informed me that when the canals of the teeth could be wiped dry it might be considered that the teeth were cured and could be filled; but that state of things did not arrive, and the young lady now wears four porcelain incisors attached to a caoutchouc plate.

I am aware that it is claimed that dead and diseased bone tissues exist at the end of roots thus affected, and that these remedies dissolve the bone and excite a healthy action in the surrounding parts. I am also aware that surgical treatment has been advocated and clinics given, showing how, by the use of the bur, or by incisions to the apex of the root, the "dental secreting sac" may be destroyed and the dead tissue removed. I have followed the advice of men long in years and experience, and extracted teeth, cut off the end of the root, filled the canals and put them back, and thus the supposed evil existing at the end of the root has been eradicated and a healthy reaction set in. All these well-intended but misdirected efforts have failed, because of a misconception of the trouble. I draw these conclusions both from the results of the foregoing treatment and the fact that in ninety-nine cases out of a hundred the extraction of the teeth thus involved would cure the trouble in the gums without treatment. This at once gives an idea as to the exciting cause of cold as well as acute alveolar abscess. Of the many improvements and advancements in various directions of which we can boast, the fact that though a tooth may lose its central vitality, become foul beyond olfactory endurance, and, as regards its cleanliness, only to be compared with dead men's bones, and yet be cleaned, disinfected, and set to work in one's mouth—this is not the least of that which has been accomplished.

To bring the subject clearly before our minds, let us for a few moments consider the tissues of a living tooth and their relations to each other and the surrounding alveolus, that we may more clearly understand what takes place when the central nerve-supply dies or is destroyed.

We will suppose that a vertical section has been made of a central or cuspid, a portion of which is represented in plates 1 and 2. From within outward, we have, first, the pulp and layer of odontoblasts, with filaments running from the odontoblasts into the tubuli which permeate to the inter-globular or granular spaces. These tubuli carry the nerve fibrils and what nourishing matter the dentine receives from within outward, at least as far as the inter-globular spaces; whether they continue further is not now clearly shown, but it is evident that these nerve fibrils anastomose with those from the cementum and peridental membrane freely at this space, though in all cases one would not be justified in saying that the nutriment supplied to this portion of the tooth comes from without, and not from the pulp.

Inasmuch as the cemental structure is less dense and more abundantly supplied with living or soft tissues, we are justified in the conclusion that at least the inter-globular spaces receive the greater supply, if not the whole of their nourishment, from without.

Covering the cemental structure, the peridental membrane furnishes the connection between alveolus and cement, and a medium of connective tissue through which the efferent and afferent nerves and vessels pass. We have before us, then, the living dental matrix as at present commonly described in Dental Anatomy. As the lime-salts of a tooth do not die and enter into active decomposition with the soft tissues within any reasonable amount of time, their consideration is not necessary as a factor in diseased teeth. The mature, well-vitalized tooth-organ, is capable of repair when subject-

Fig. 1.

Fig. 2.

ed to injury, provided the cause of wasting tissue is mechanically or chemically removed; just the limit of this power cannot be determined in a given case, nor can we say just when this power may or may not be present. It is very evident that at times and in certain cases, a slight amount of mechanical or chemical interference with the pulp of a tooth will destroy its function, and render desired reparation impossible. There seem to be marked exceptions, however, where nature alone has not only overcome the destroying agent, but protected the exposed pulps with new formations of tissue.

Our success in assisting nature in these processes has thus far been questionable, as the many cases of diseased and septic teeth testify. It may be that exposed pulps at some future time will be capped to live, but with the present means at command they are, for the most part, capped to die, and with the increased possibility of retaining a higher state of vitality I believe it is better practice to destroy and remove a pulp than it is to cap, with the chances of death and disease which so frequently follow. The tooth organs that have lost their central nerve and vessels are called "dead teeth." Every one knows that if a thing is dead it has not life. A tooth that has lost its central nerves and vessels is still nourished throughout its periphery. It cannot then be called dead, and if such a tooth be treated in a way to retain the highest possible benefits from this source of support, it will be tolerated by the surrounding tissues, and prove serviceable for many years.

For convenience, I shall divide my subject into four classes :—

1. Teeth with central nerves and vessels extirpated, and free from internal and external septic infiltration, with the peridental membrane performing its normal function.

2. Teeth with their central nerves and vessels destroyed, and their decomposed remains unremoved, and septic infiltrations extending to the peridental membrane, which becomes inflamed at the slightest depressing influence on the general system.

3. The death of the central pulp, followed by putrefaction and infiltration of all the dentinal tissue, the cemental tissues infected, inflamed, thickened, and a connective tissue wall formed protecting the deeper parts of the alveolus. This wall is usually located at the foramina at the apex of the root, and is generally known as the "secreting sac," leading from which is the fistulous passage opening to the surface offering the least resistance of tissue. When this condition is allowed to continue for years, the root membrane is destroyed, and even the root itself is absorbed wholly or in part.

A tooth becomes dead when it loses its membrane as well as its central pulp. These teeth I have designated as the 4th class. Their destruction is rarely due to the death of the pulp by internal causes, but more especially from outward wasting of tissues, from calculus or diseased alveolar tissues, when without support such teeth may be called dead, irritating, foreign bodies.

In the four classes of diseased teeth mentioned we observe variable conditions, any one of which of the milder forms may be but the progressive stages of the same trouble, which if not arrested will lead to the loss of that tooth, if not to more grave results. A tooth if deprived of its central nerve and blood supply resists destructive agencies of decay by its chemical integrity, in so far as this integrity is not overcome by what may be called "greater chemical affinities." As it becomes septic, within the pulp-chamber, its destructive tendencies are two-fold; it is "a house divided against itself," and the only agents necessary to support and carry on the destruction of the tissues are water and air. The products of this change, as we find them, are derived primarily from the pulp matter, but later from the breaking down of the organic matter of the tubuli. The main portion of the pus, however, in the progressive stages, is derived from the diseased or infected tissues immediately at the apex of the root, the fistulous opening usually appearing on the surface of the gum, carrying or furnishing vent to the matter and gas to which it augments. The generation of gases accompanying the breaking down of the dental tissues is, for the most part, the same as those generated from decomposing organic bodies whose constituents are largely of phosphorus matter, as the brain, bone or fish. This gas I apprehend to be phosphoretted hydrogen. It is found also in closed cavities of decaying teeth, and is significant of decomposing organic and inorganic tooth structure into ultimate proximate principles, as lime, magnesium, phosphoric acid, or phosphorus, and probably the re-formation of other substances, as in the case of phosphoretted hydrogen. There are three known compounds of phosphorus and hydrogen—($PH^3$) ($PH^2$) ($P^2H$). I cannot determine at present whether one or all are present, but am prepared to say the first named ($PH^3$) has been detected. And here I take the liberty of calling attention to a paper previously published in the INDEPENDENT PRACTITIONER by me (see Vol. VII, p. 1), giving experiments to show the presence of free phosphoric acid in dental caries, derived from the decomposition of the proximate principles of teeth (phosphate of lime and magnesia), as it is seen that further proof of this decomposition appears in the presence of phosphoretted hydrogen in caries as well as in decomposing pulp matter.

If we are to weigh the questions of life or the death of an organ, our attention is first called to known arrangements of tissues, that involved in death and that living, and we draw our conclusions as to the vitality upon which rests our chief reliance for support; we look at the condition of a tooth without central blood-vessel or nerve supply, and we see at once that the only reliance for vital support is from the cemental tissues, and all treatment must be directed to preserve these tissues from the destructive processes progressing in the dentine. When once this infectious matter has invaded the pulp chamber and tubuli, the tooth becomes ever after an easy prey to these changes, and in no way can it be kept so free from septic influences as to prevent it from becoming septic.

I am strongly of the opinion, therefore, that great care should be exercised in the capping of exposed, or filling very near diseased, pulps. Barring inaccessible root-canals, which are very rare, the weight of evidence is in favor of destroying rather than capping a pulp. Good judgment, reinforced by the knowledge of a defective constitution and the general depleting effects of certain conditions and influences in life, will often decide what course it may be necessary to take in a given case.

But for various reasons teeth do die, and we find dead pulps resulting from numerous causes, and though not always septic, they are liable to become so if at all exposed. It is well known that a devitalized pulp may not cause any irritation, either during death or for years after, but this cannot be regarded as evidence that such teeth should not be disinfected, cleansed and sealed up, but should be regarded rather as an indication of the power of the general health to take up or absorb dead matter. Such power, however, may or may not be present at different periods of life. For instance, a tooth may remain quiet for years, and then suddenly enter upon acute inflammation and terminate in a chronic cold abscess. The poisonous matter generated from the dentine having infected or poisoned the soft tissues, the system no longer able to carry on the absorbing process, it expels the matter and provides for future accumulation by a fistulous opening and a connective tissue wall around the infected parts.

We have thus described the second class of teeth named, and indicated the progressive stages of septic influences which render the tooth more and more irritating to the surrounding parts. We often find that the fistula, if probed, leads to a large cavity immediately at the end of the root, and the root, denuded of its membrane, furnishes a portion of the wall to the cavity named. It has often been stated that in connection with the above described condition the primary exciting cause in many cases is superseded by a secondary cause. The alveolus is infected, dies or becomes diseased from the infectious matter from the tooth, a condition of necrosis existing which requires special attention and treatment independent of the tooth. During the time I have been in practice I have seen but one case of the kind mentioned, and, as has been said, I am of the opinion that the cases of diseased alveolar abscess referred to that cannot be cured by the extraction of the teeth involved are rare, showing that if the teeth can be rendered aseptic the alveolus and tissues about such would return to a condition of health and usefulness.

I think I have made it clear that I am in favor of securing the best possible condition of dentinal tissue where there is the least possibility of their becoming pulpless, either by accidental, chemical, or by any other means. By observing the cuts, we see a somewhat enlarged or thickened cemental covering, in which case the possibilities of life after the death of the pulp are quite considerable. I am aware that similar thickenings of the cemental structure are classed as abnormal growths, but if observation be made it will be found that the thickness of the cementum differs largely in proportion to the dentine in different teeth, giving to some pulpless teeth a great degree of vitality.

Treatment.—When the cause of the trouble and the conditions of tissue are well understood, the question of treatment is often very simple. With the present difficulty and the means at hand, barring mal-formation of the roots, the chances of success are more than equal to any operation we are called upon to perform. As in all diseases of the system, if our patient is strong and vigorous, no matter how long the abscess has existed, fifty per cent. of success is assured. On the other hand, desired results are not so speedily obtained in more delicate or enfeebled health. In all cases, relief from pain is the first step in treatment; often an aching, exposed pulp may, by the use of narcotics, be treated into a state of rest, and the pulp destroyed.

For the destruction of pulps, I have found for the present, nothing that meets the wants of the case so well as arsenious acid, though I am of the opinion that a better preparation can be prepared. That used by me is composed of equal parts of arsenious acid and the acetate or sulphate of morphia, made into a paste with carbolic acid or creosote. Right here is danger. Many inflamed peridental membranes are caused primarily by arsenic preparations getting through the apex of the root and creating dead tissue and aseptic influences. It is better to cause the patient a little pain than to run the risk of a diseased membrane. I have used nitrate of silver with very favorable results. It does not cause pain, and its destruction of tissue is immediate.

With the usual means, the roots are cleared of all the pulp matter possible, dried with hot air until there is no moisture; not only the pulp canals, but the dentinal tubes should be dried as far in as possible, after which the alcohol may enter even to the inter-globular spaces. In filling, I make use of the well-known preparation of gutta-percha and chloroform. In the treatment of abscessed and septic teeth, of course a long cleansing process is necessary. I strongly oppose the method of opening through the apex of the root, as by so doing there is great liability of further infecting the tissues by means of the infected drill, though I use the drill to get a free opening as near the apex as possible. This done, the canals are washed out thoroughly with warm water and dried with hot air, after which it is saturated with a solution of strong bichloride of mercury (five per cent).,* the opening in the crown being stopped

* At the meeting at Newark, N. J., before which this paper was read, my at tention was called to the solubility of bichloride of mercury in water. Three years previous, I had prepared for me a bottle containing a strong solution of the bichloride, with directions on the label to prepare any strength down to one part in three thousand. I find the label reads one in twenty, not twenty per cent.

The strongest solution used by me is, therefore, five per cent., and this for with gutta-percha. This is repeated once in three or four days until there is no smell of phosphoretted hydrogen, and I am satisfied that the tooth is thoroughly aseptic, when it is sealed up and filled as in the previous case.

After a portion of the peridental membrane has been destroyed, is it possible to render the cemental structure so aseptic that the alveolar tissues will harden about the exposed surface without irritation? Of course there is not that assurance of success as in the previous cases, but in the mouth of the average person of health, the bichloride treatment should be tried both outside and in. I have been surprised at the results of the cases treated; out of three, two have been successful; the other was a case where I could hardly expect success; so extensive was the trouble that three of the superior centrals had their roots partially absorbed, and the only course seemed to be extraction, which, after the operation, I did not regret at all. Although badly diseased and absorbed, the alveolus healed very quickly without treatment.

It is hardly necessary for me to say that my treatment for the fourth class of teeth named is extraction, as they are indeed "dead teeth" and "foreign irritating bodies."

---

treating dentinal tissues, not soft tissues, or hard tissues where it would be liable to reach the soft tissues. For the latter use, the strongest used would be one part in two hundred.

There are two chlorides of mercury; First, calomel, sub-chloride, or protochloride of mercury, or mercurous chloride. ($HgCl$.) This differs from bichloride in not being soluble in water.

Second, Corrosive sublimate. Chloride of mercury. Bichloride or per chloride, or mercuric chloride. ($HgCl^2$.)

This dissolves in three times its weight of boiling water, but requires sixteen parts of cold water to one of bichloride.

Muriate, or chloride of ammonia, is used to render bichloride of mercury more soluble in water. It does not change the chemical composition of the bichloride, and no new compound is formed, but acts simply by its presence. The bichloride may then be secured in any strength desired.

I cannot see that the ammonia chloride thus used has any injurious effects on the tooth-structure, or at all hinders the antiseptic action of the bichloride.

## Figures and Tables

**Fig. 1. f1:**
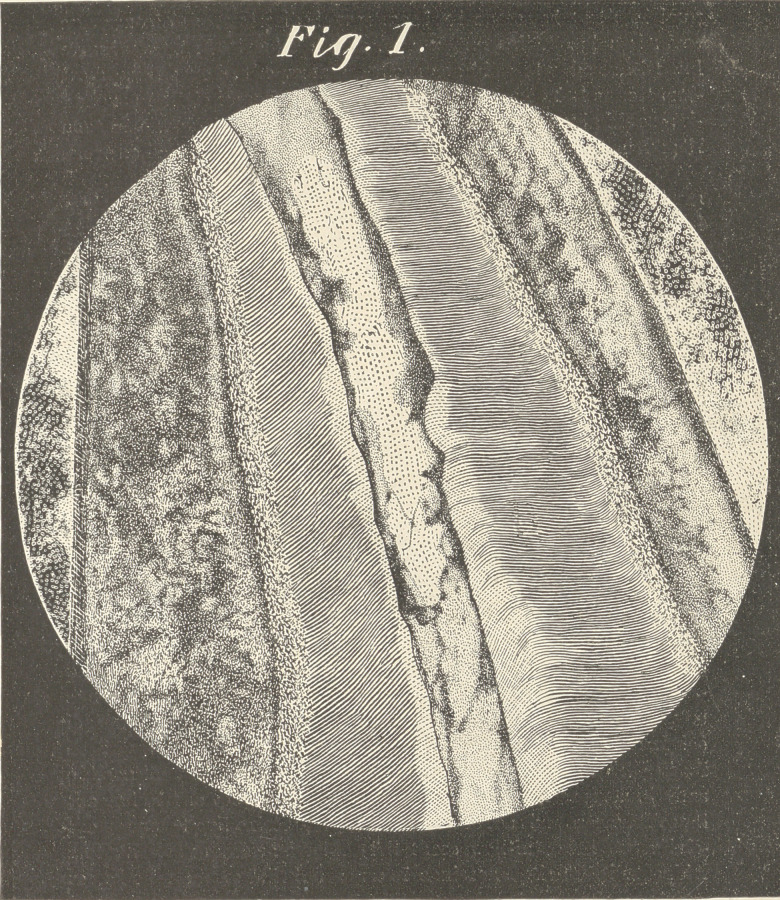


**Fig. 2. f2:**